# ‘Those Were Smiling Eyes’: Patients' Experiences and Perceptions of ICU Health Care During Pandemics

**DOI:** 10.1002/nop2.70335

**Published:** 2025-10-22

**Authors:** Ilenia Piras, Maria Francesca Piazza, Marta Sarritzu, Cristina Piccolo, Gabriele Finco, Maura Galletta

**Affiliations:** ^1^ Clinical Trials Sector, General Affairs ‘A. Cao’ Microcythemia Hospital, ASL Cagliari Cagliari Italy; ^2^ University Hospital of Cagliari Monserrato Italy; ^3^ University of Cagliari Monserrato Italy; ^4^ Intensive Care Unit SS. Trinità Hospital ASL Cagliari Cagliari Italy; ^5^ Intensive Care Unit University Hospital of Cagliari Monserrato Italy; ^6^ Department of Medical Sciences and Public Health University of Cagliari Monserrato Italy

**Keywords:** COVID‐19, healthcare, intensive care unit, nurses, pandemics, patient experience

## Abstract

**Aim:**

The study aim is to explore the experiences of patients who were admitted to intensive care units due to severe SARS‐CoV2 infection and their perceptions regarding the health care they received. This is important to provide appropriate support to the patients and prepare organisations for future pandemics.

**Design:**

Qualitative investigation with phenomenological approach.

**Methods:**

A semi‐structured interview with 27 patients discharged from intensive care units was performed. The research was carried out from May to December 2021 in Italy. Participation was voluntary, and informed verbal consent was obtained from all participants after a full explanation of the study objectives.

**Results:**

Thematic analysis of participants' interviews revealed five core themes related to their ICU hospitalisation experiences and perceptions of care: (1) Quality of received care, (2) Critical care issues, (3) Personal protective equipment and patient–healthcare professional interaction, (4) Relationship with nurses and (5) Strategies to ensure communication. The results show that despite barriers due to safety devices, patients with COVID‐19 felt supported and cared for by healthcare professionals in several aspects during hospitalisation. However, post‐discharge care programs are needed to reduce the long‐term effects of the disease and provide more patient‐centered care in the intensive care units and during future outbreaks. The study results offer interesting insights for improving practice in intensive care units and patient care in the event of future pandemics.

## 1 Introduction

The emergence of COVID‐19 has posed an unprecedented challenge to healthcare systems worldwide. The spread of the SARS‐CoV‐2 virus resulted in complex human disease, mainly characterised by acute respiratory illness of varying severity (WHO 2024). The highly contagious nature of the virus and the high mortality rate immediately imposed severe restrictions on social interactions and disrupted the existing organisation of health care. Health systems had to cope with suddenly large numbers of patients, both locally and in hospitals. As a result, existing inpatient wards in hospitals were reorganised, new beds were activated, and more intensive care unit (ICU) beds were needed to care for patients with severe complications.

ICUs have become critical battlegrounds in the fight against this novel and highly contagious virus. Patients admitted to the ICU with COVID‐19 often experience a complex and frightening hospitalisation due to the severity of their illness and the unfamiliar high‐tech environment (Sahoo et al. 2020). Patients are often heavily sedated and dependent on mechanical ventilation (Cederwall et al. 2018), which further impairs their ability to communicate and understand their surroundings. The need for a massive use of personal protective equipment (PPE) to reduce the contagion may have a negative impact on care, by creating a real barrier and hindering communication between professionals and patients (Aengst et al. 2022). Also, the global shortage of health care professionals (HCPs) could put a strain on professionals who are already under pressure to care for patients during the pandemic as well as respond to the normal care of non‐COVID‐19 patients. In addition, the introduction of strict restrictions to limit the spread of infection led to the suspension of visits to hospitalised patients, which can contribute to feelings of isolation and loneliness (Xu et al. 2024). In this challenging situation, care was mainly focused on the acute aspects of the disease. Of the nursing activities provided, most were technical in nature at the expense of those of a holistic nature (Fernandes et al. 2022; Imam et al. 2023), with the result that a significant number of nursing interventions were not provided (Fernandes et al. 2022).

Understanding the lived experience of patients during their ICU admission is critical to providing appropriate support to patients with COVID‐19 or other infections and to preparing for future pandemics. Although quantitative studies have provided valuable data on clinical outcomes and resource allocation during COVID‐19 (Silva et al. 2020), they cannot capture the richness of the patient's subjective experience, which can better explore patients' perceptions of their hospitalisation and the quality of care they received, and provide insights for improving patient‐centered care practices. Research shows that the lived experience of patients with COVID‐19 admitted to the ICU often extends beyond the physical challenges of the disease to include the psychological challenges of isolation, fear of the unknown and death (Piras et al. 2022). Research on hospitalisation experiences in general emphasises the importance of factors such as a sense of control and clarity of communication in determining patient well‐being (Oben 2020). Previous research suggests that positive nurse–patient relationships in the ICU are crucial for promoting feelings of security and influencing clinical outcomes (Happ 2021). However, despite the growing literature on the experiences of patients in intensive care units, there is still little research aimed at gaining an in‐depth understanding of the healthcare experiences of Italian patients admitted to intensive care units for severe SARS‐CoV‐2 infection. Our study is important because Italy was the first European country to be affected by the COVID‐19 pandemic, with a very high mortality rate (Istituto Nazionale di Statistica [ISTAT] 2020), which led the national health service to a sudden and far‐reaching crisis. This situation created a peculiar care context, still little explored from the point of view of direct patient experience. This qualitative study aims to address this gap by providing an in‐depth understanding of the experiences of patients who survived admission to the intensive care unit for SARS‐CoV‐2 infection, based on their memories of hospitalisation. Precisely, the study is intended to explore patients' experiences of perceptions of care received and interaction with professionals and experiences of communication and connection with the outside world.

## 2 Methods

### 2.1 Study Design and Procedure

The study includes a qualitative investigation via semi‐structured interview to patients discharged from intensive care units (ICU) following. SARS‐CoV‐2 virus infection. The research was performed from May 10th to December 15th, 2021, in Italy. Potential participants were identified based on their experience of hospitalisation in ICU due to COVID‐19, which they spontaneously told reporters, on television, on social media. Recruitment was through research in online newspapers and public social network pages where participants gave interviews or wrote about their experiences with the illness and hospitalisation. To enrol, patients had to agree to participate and provide written informed consent.

People were contacted through available phone numbers or e‐mail addresses. When phone or e‐mail was not available, secondary information such as job, profession and city from news reports was used to continue the web search. A total of 40 potential participants who met the inclusion criteria were listed. People were contacted by phone or email to propose participation in the study.

### 2.2 Tool

Based on the study purpose, a semi‐structured interview guide with four main questions (Table 1) was developed and adapted from available literature (Sahoo et al. 2020; Sun et al. 2021). The guide aimed to capture patients' experiences of care and interaction with HCPs during their time in the ICU, as well as their experiences of communication and connection with the outside world. Interview questions were used flexibly to ensure the naturalness of the conversation. During the interviews, stimulus‐questions such as ‘Can you explain further?’, ‘What does that mean?’ or ‘Please give an example’ were used to clarify or elicit further details. At the end of the interview, all the participants were asked if there was anything they wished to add or recount in relation to their lived and current experience.

**TABLE 1:** Interview guide.

Questions guide
Perception of care and interaction with HCPs
(1) How did you experience about caring provided during hospitalisation (in ICU)?
(2) What was your perception about healthcare professionals (in ICU) who interacted with you exclusively while wearing suits, visors and lots of protective gear?
(3) How was your experience with health care professionals during hospitalisation (in ICU)?
Communication and connection with the outside world
(4) How did you communicate with the outside world (relatives, friends, …) during hospitalisation (in ICU)?

### 2.3 Data Collection

Based on the criteria established for recruitment, we developed a list of 40 potential participants. Of these, 6 individuals were untraceable or did not answer the call, 7 refused to participate in the study. Finally, 27 persons joined the study. At first, participants were contacted for presenting the research purpose and choosing with them the most suitable interview method (e.g., via phone or video call). The researchers were available to provide any clarifications about the study and used methods.

Interviews were audio‐recorded with an average duration of 30 min. The researchers were trained in narrative interviewing techniques prior to data collection. Semi‐structured interviews were conducted with participants to elicit detailed narratives. Verbatim notes were taken throughout the interviews to document key points for later analysis. Interviews continued uninterrupted until data saturation was reached, indicated by the absence of new information from subsequent interviews (Merriam 2009).

### 2.4 Ethics Statement

Email addresses and phone numbers of patients who were interviewed by the media were publicly available. Informed written and verbal consent was obtained from all participants after a full explanation of the study objectives. Participation was voluntary, and interviews were recorded with participant consent. Participants were assigned unique sequential identification codes to ensure anonymity. The study adhered to the ethical principles outlined in the Declaration of Helsinki and the General Data Protection Regulation (EU) 2016/679 (GDPR). Participants were explicitly informed of their guaranteed anonymity in accordance with Italian privacy law (Decree No. 196/2003), as well as their right to withdraw from the study at any time without penalty. The local Ethic Committee approved the research project with protocol number NP/2023/495.

### 2.5 Analysis

Qualitative data analysis was used following the method of Colaizzi (Colaizzi 1978). All interviews were transcribed verbatim, including colloquialisms, tone variations and silences, in order to fully capture the lived experience.

#### 2.5.1 Independent Analysis

First, each researcher independently read the interview transcripts to familiarise themselves with the data. They then proceeded to independently code the transcripts focusing on identifying key statements and concepts relevant to the research objective. A combined inductive and deductive coding approach was utilised; new codes emerged during analysis to capture the nuanced aspects of the data.

#### 2.5.2 Collaborative Analysis and Triangulation

Once individual analyses were complete, the research team collaboratively analysed the initial codes. Through group discussion, the codes were then grouped into larger themes. This process was highly iterative, with themes continually refined as researchers revisited the transcripts to ensure they precisely captured participants' experiences. Ultimately, shared major themes emerged, which were then reviewed for clarity and consistency, and each was clearly defined and named. Finally, the results were documented with sample quotes for each theme. This collaborative method allowed themes to develop from intense group discussions, minimising individual interpretations and researcher bias.

#### 2.5.3 Rigour and Reflexivity

To further ensure methodological rigour and minimise bias, researcher reflexivity was encouraged. The researchers were aware that the clinical experience of some of team members with Sars‐Cov‐2 patients might influence the interpretation of the data. To mitigate the influence of pre‐understandings, open discussion among team members and triangulation of data were stimulated. Field notes containing interviewer reflections were discussed to promote self‐reflexivity and audits were conducted among team members throughout the research process.

## 3 Results

### 3.1 Demographics

The study included a sample of 27 participants living in Italy. Participants ranged in age from 37 to 75 years, with an average age of 56.85 years. The majority of the participants (67%) were male. Eight men were younger than 50 years and four women were older than 60 years. Regarding ICU length of stay, 18.5% (5 of 27) of the participants were hospitalised for 10 days or less, 37% (10 people) were admitted for 11–30 days, and 41% (11 people) were admitted for more than 1 month and up to 2 months. The median of total length of stay was 47 days, with a range of 18–120 days. Detailed results are shown in Table 2.

**TABLE 2:** Interviewers' characteristics and length of stay (LoS).

Participant ID	Gender	Age	ICU LoS (Days)	Total LoS (Days)
P1	M	56	8	22
P2	F	46	11	30
P3	M	48	19	20
P4	F	75	6	18
P5	M	65	52	67
P6	M	71	42	50
P7	F	58	22	43
P8	M	48	16	33
P9	F	65	10	47
P10	F	59	71	90
P11	M	69	32	96
P12	M	69	7	34
P13	F	37	17	24
P14	M	54	40	89
P15	F	63	26	51
P16	F	52	18	66
P17	M	41	18	21
P18	M	38	10	45
P19	M	48	45	90
P20	M	49	34	90
P21	M	57	19	41
P22	F	75	42	90
P23	M	70	54	120
P24	M	66	40	55
P25	M	65	22	40
P26	M	43	48	108
P27	M	48	21	38

### 3.2 Main Themes and Subthemes

Thematic analysis of participants' interviews revealed five core themes divided into two main categories: (a) patients' experiences of care and interaction with HCPs during the ICU hospitalisation, and (b) patients' experiences of communication and connection with the outside world. The first category includes themes from 1 to 4 related to: (1) Quality of received care (further divided into two subthemes); (2) Critical care issues (including three subthemes); (3) PPE and patient‐healthcare professional interaction (including four subthemes); and (4) The relationship with nurses (divided into two subthemes). The second category includes the last theme related to: (5) Strategies to ensure communication (further divided into three subthemes). Details of these themes are shown in Table 3.

**TABLE 3:** Main themes and subthemes emerged from the interviews.

Theme	Subtheme	Interviewee
Category a: Experiences of care and interaction with HCPs		
1. Quality of received care	1.1 Professional and human care	P1, P5, P7, P21, P24, P25
	1.2 Perception of continuous change in caring	
2. Critical care issues	2.1 Practical issues due to the use of personal protective equipment (PPE)	P1, P4, P7, P8, P11, P13, P14, P15, P16
	2.2 Shortage of personnel	
	2.3 Lack of a post‐Covid‐19 path	
3. PPE and patient‐healthcare professional interaction	3.1 Awareness of the need to use PPE	P2, P3, P4, P6, P8, P10, P12, P17, P19, P21, P22, P27
	3.2. Feeling of loss and confusion	
	3.3 PPE as barriers in verbal communication and importance of nonverbal communication	
	3.4 Beyond the mask: the use of eyes to communicate emotional closeness	
4. The relationship with nurses	4.1 Nurses as family	P8, P11, P18, P23, P24
	4.2 A ‘special’ connection with nurses	
Category b: Experiences of communication and connection with the outside world		
5. Strategies to ensure communication	5.1. Methods of communication between healthcare professionals and patients	P1, P5, P9, P10, P16, P20, P26
	5.2. Communication between professionals and patients' families	
	5.3 Using technology to communicate with loved ones	

#### 3.2.1 Category a: Experiences of Care and Interaction With HCPs

##### 3.2.1.1 Theme 1. Quality of Received Care

This theme highlights both the positive aspects of care, which focus on the professional and human qualities of staff, and the challenges posed by a rapidly evolving pandemic situation with limited knowledge of the best treatments.

###### 3.2.1.1.1 Sub‐Theme 1.1 Professional and Human Care

In the following interviews, patients recognize the skill and dedication of all HCPs in providing critical care and assistance in a difficult situation by highlighting the professionalism and humanity they received during their stay in the intensive care unit (ICU). In particular, patient P21, while acknowledging the flaws and weaknesses of the health care system, emphasizes the continuous dedication and professionalism of HCPs, highlighting their ‘excellence’ and ‘incredible’ commitment. Participant P24 emphasizes that the good care received is not only due to the clinical interventions, but also to the HCPs' ‘human aspect’. Again, patient P25 uses adjectives such as ‘incredible’, ‘exaggerated’ and ‘crazy’ to describe his appreciation for the HCPs' care efforts during the pandemic.

I noticed the excellence of Italian healthcare, in the healthcare professionals who working there. The system in general, in this pandemic, has also shown many flops and weaknesses, but the substantial healthcare assistance is provided by people, let us not forget, and it has been excellent. (P21)

The healthcare was good, not only because they saved me and therefore it is easy to say, but precisely from a human point of view. (P24)

The healthcare (…) was incredible, exaggerated, crazy… what nurses, physicians, healthcare assistants did…. I do not know how they did it. (P25)

###### 3.2.1.1.2 Sub‐Theme 1.2 Perception of Continuous Change in Caring

Interviewees are aware of the rapidly changing treatment scenario for COVID‐19 influenza. They recognize the existence of differences in treatment protocols over time and the lack of established protocols at the beginning of the pandemic. Participants also report information dissonance and uncertainty about the best course of action (e.g., vaccines), revealed from their online information search. These aspects are highlighted by participants P1, P5 and P7:

We were in October [2020], and after a couple of months, when I spoke to other people, the treatment was different; everything was evolving. When I entered, they gave me about twenty pills at a time, each different from the other. (P1)

(…) There were no specific treatments; it was all based on the experiences of one year, maybe one year and a half. I went to read up on the internet, and even there, in short, the news were not so good. (P5)

(…) There is still much insecurity, even now, for the vaccine. I should get the vaccine, and everyone has his/her say about it. I have heard from three different people and even here [among HCPs], some say to wait one year and others say to do it because it is safer than not doing it. In short, everyone has his/her say. (P7)

##### 3.2.1.2 Theme 2. Specific Critical Care Issues

This theme explores the specific critical care issues related to the pandemic time. The sub‐themes highlight challenges related to personal protective equipment (PPE), HCPs shortages and the lack of a post‐COVID‐19 care pathway.

###### 3.2.1.2.1 Sub‐Theme 2.1 Practical Issues due to the Use of PPE

The interviews emphasize the challenges posed by the use of PPE. Participant P1 highlights that the fogging up of visors hindered clinical procedures and fluidity of movement. Participants P4 and P14 describe the physical and psychological discomfort experienced by HCPs due to the intense heat and the cumbersome nature of PPE. Phrases such as ‘one doctor fainted on my bed’, ‘it took 10 to 15 minutes to get dressed and the same to undress’ and ‘they didn't have a good time’ highlight the physical and emotional stress faced by HCPs.

Several times they [HCPs] had to interrupt the blood gas, that is quite difficult, because their mask fogged up. In my opinion, they looked for each other, and it was more uncomfortable for them than for patients. (…). (P1)

(…) It was difficult to hold all these barriers. They [HCPs] were sweating, one doctor fainted on my bed (…). (P4)

They did not have a good time; they had many problems because, unfortunately, they were the first to die of heat. It took 10 minutes or a quarter of an hour to get dressed and the same to undress. They worked gruelling shifts. In short, in my opinion, they, too, did not experience this period very well. They had many difficulties. (P14)

###### 3.2.1.2.2 Sub‐Theme 2.2 Shortage of Personnel

Patients described ICUs as overworked and understaffed to deal with the peak of the pandemic. Patient P7 tells of marked staff shortages during holiday periods (e.g., Christmas) and highlights an excessive workload as treatments started very early in the morning and ended very late in the evening. Patients P8 and P16, while highlighting the preparedness and competence of HCPs, emphasise the inadequate ratio between the number of professionals and the amount of patients to be managed in the COVID‐19 emergency.

(…) They were overworked. Then it was the Christmas holiday [2020], so they probably also had reduced staff. (…) they did the therapies until one or two o'clock in the morning, and the blood samples instead already at five o'clock in the morning. Furthermore, they went around the beds until one and two o'clock in the morning, exhausted. (P7)

(…) The second one [the hospital in which the patient was admitted] was just fine, but lacked staff; in the sense that the staff was there, they were trained, prepared, and ready to intervene, but they were lacking compared to the wave of sick people that overwhelmed them. We needed many more nurses, physicians, specialists, and professionals from that point of view. (P8)

My understanding was that the situation was a total emergency anyway, there were no doctors, I saw them once a day. (P16)

###### 3.2.1.2.3 Sub‐Theme 2.3 Lack of a Post‐COVID‐19 Path

A central theme of the interviews is the perceived lack of adequate care after discharge. Participants express frustration with the absence of consistent follow‐up and support after hospitalisation or post‐COVID symptom management. Participants P11 and P13 place emphasis on human connection, claiming that after discharge there is no one to give emotional or practical support to cope with the complexities of post‐COVID life. Patient P13 also highlights the lack of a standardised post‐COVID pathway, suggesting the need for a more coordinated and comprehensive approach to the management of long‐Covid symptoms. Patient P15 particularly expresses the feeling of loneliness by claiming that s/he did not receive support even from his/her family physician.

(…) After the disease, there is no one to give you advice, help, anything. By the way, they [HCPs] never called to know if you were dead or not dead, how you were…. (P11)

(…) The aftermath, the post, should also be managed better, especially for those who have had an experience in intensive care unit and those with long Covid syndrome. In the sense that even if you go for a visit because you have some symptoms (…), you have to pay for it yourself most of the time. Instead, in my opinion, there should be a post‐Covid path that some hospitals have activated… it should also be present in small communities (…). (P13)

When I returned home [after hospitalization] I had difficulty because no one looked me after. I still presented all the hospital papers to the family physician. Then there was chaos, unfortunately, at that time, but I had many difficulties afterward. I felt alone. (P15)

##### 3.2.1.3 Theme 3. PPE and Patient‐Healthcare Professional Interaction

This theme presents the patient experience in relation to professionals who use PPE.

###### 3.2.1.3.1 Sub‐Theme 3.1 Awareness of the Need to Use PPE

Participants show an understanding of the importance of using PPE to protect HCPs. They recognized the inherent danger of the virus and the need to take these measures to reduce the risk of infection.

I perceived that the healthcare professionals had to protect themselves, and they did a backbreaking job. They had to protect themselves because (…) they were all young people, young physicians… so they were right to protect themselves. (P4)

I perceived that the professionals had to protect themselves from something very dangerous that I had [as they used the protective suits]…. (P8)

At first it was strange [to seeing healthcare professionals with PPE], but then you understand that there is this need, that there is Covid―this critical thing―that you have to protect yourself and use masks…. (P17)

###### 3.2.1.3.2 Sub‐Theme 3.2 Feeling of Loss and Confusion

Although patients recognised the protective importance of PPE, it was difficult to identify and differentiate HCPs. Participants P2, P6 and P21 express the concept of loss of identity. They tell of a sense of anonymity and lack of recognition of HCPs. They describe the feeling of interacting with faces rather than people, generating confusion and disorientation in participants. They struggled to recognise and identify specific HCPs (nurses, physicians, or speciality).

They [HCPs] were all the same to me. I didn't know who they were, if physicians, nurses… they were almost all the same; you did not distinguish them (…) at all. (…) You saw their faces, but if you did not remember the name, you did not remember who he/she was…. There was confusion in this sense. (P2)

Well, as a form of anonymity, I had difficulty recognizing them [HCPs]. (…) then one thing that annoyed me is that there was no element can put you in a position to identify the physician, the nurse, and the healthcare assistants; they are all anonymous. (P6)

At first the feeling was one of bewilderment. I didn't know who I was talking to, I didn't know if the nurse in front of me was the one from the day before, I didn't know if the doctor was the one who admitted me, if he/she was a cardiologist or a pulmonologist. In short, it was chaos. (P21)

###### 3.2.1.3.3 Sub‐Theme 3.3 PPE as Barriers in Verbal Communication and Importance of Nonverbal Communication

Participants expressed a sense of detachment with HCPs due to the impersonal nature of PPE. Patient P10 recognizes that although PPE is necessary, it can create a barrier to human contact. Patient P12 strongly reiterates this concept by stating that PPE makes HCPs ‘all look like the same.’ However, participants highlight HCPs' efforts to maintain a human connection through using gestures and tone of voice. Patient P22 points out that HCPs used their eyes to communicate warmth and empathy, thus reducing emotional distance despite physical barriers.

(…) The devices were a detachment but at the same time a necessary barrier. In their voice and gestures they [HCPs] let such a humanity shine through that only physical contact could give. (P10)

they [HCPs] all looked like the same, one taller, one shorter, but they were all the same. (P12)

(…) The overalls and the visors created distance, but they [HCPs] did everything to make us not feel such a distance, especially using their eyes; with the eyes they talked, smiled and joked. (P22)

###### 3.2.1.3.4 Sub‐Theme 3.4 Beyond the Mask: The Use of Eyes to Communicate Emotional Closeness

Patients emphasise the crucial role of eye contact in human communication when facial expressions are covered by PPE. Patient P3 tells about how HCPs are able to effectively convey emotions such as serenity and compassion through their eyes. This aspect is reinforced by patient P19, who reports that the limitations imposed by PPE led to rediscovering the power of eye contact. By focusing solely on the eyes, s/he was able to perceive a deeper level of human connection and understand emotions more deeply. Participant P27 uses the word ‘charity’ to describe the emotions conveyed through the eyes of HCPs, thus perceiving a sense of compassion and altruism despite the difficult circumstances.

Only from the looks you could perceive closeness [by the HCP] (…) only the eyes you could see because of all the harness they were wearing. However, from the looks they managed to convey serenity. (P3)

Not seeing face expressiveness could initially seem like a handicap; I couldn't understand other people's emotions. But then I started looking at the eyes, and the eyes spoke much more than the face; you rediscover the beauty of looking into people's eyes and being able to understand many things just from the gaze. (P19)

You could only see their eyes. I… well… (silence)… I saw charity in those eyes. I saw charity. So it was enough for me to see the eyes, I was not interested in seeing the face or anything else. (P27)

##### 3.2.1.4 Theme 4. The Relationship With Nurses

This theme highlights the development of close relationships between patients and nurses in the ICU during the hospitalisation.

###### 3.2.1.4.1 Sub‐Theme 4.1 Nurses as Family

Hospitalisation, particularly during isolating periods, can create a sense of loneliness and disconnection from loved ones. Nurses, through their consistent presence, compassion and emotional support, fill this void. This highlights the crucial role of human connection and emotional support, beyond the provision of clinical interventions, as emerges from patients P11 and P24:

At some point a friendship was born because they were the only people who came to visit us. They [nurses] were the only relatives, as I used to call them. They cared about us as people, not just as nurses. (P11)

I remember (…) when the nurses approached the bed and shook my hand. For me it was like my mother and father were there, a friend, a brother (…), they were like our relatives. (P24)

###### 3.2.1.4.2 Sub‐Theme 4.2 A ‘Special’ Connection With Nurses

The patients' interviews reveal a unique bond with nurses. Their narratives highlight the invaluable role of nurses as companions in adversity, and thus a sense of dependence on them as their only stimulation and contact with the outside world. Patients P8 talk about nurses as ‘precious’ persons during difficult times. Patients P18 and P23 use phrases such as ‘extraordinary relationship’ and ‘they were always there when you need them’ to emphasize the vital role played by nurses in caring. Nurses are perceived as the only source of external contact able to recognize and meet patients' needs without having to express them.

Especially the nurses were precious because they were there during the hard times. (P8)

An extraordinary relationship was arised with the nurses. We almost went through this experience together, me, in the passive part, and they in the active one, a moment in which there were only them and us patients. We had them as the only stimulus and outward contact. (P18)

You build a very strong relationship with the nurses, they are always there when you need them, and in this circumstance they also replaced our voice and our hands. (P23)

#### 3.2.2 Category b: Experiences of Communication and Connection With the Outside World

##### 3.2.2.1 Theme 5. Strategies to Ensure Communication

This theme explores patients' experiences with a specific focus on communication strategies used by health care professionals. It also explores communication between patients and their families, and the use of technology to facilitate this relationship.

###### 3.2.2.1.1 Sub‐Theme 5.1 Methods of Communication Between Healthcare Professionals and Patients

Some of the interviewees told about how they coped with communication limitations by relying on nonverbal language such as gestures and nods or by describing innovative solutions facilitated by professionals, including the use of written communication aids (alphabet charts and notebooks): The nurses were able to anticipate the patients' needs by finding timely solutions to enable them to express themselves even when they could not speak.

(…) I spoke badly and I had a mask that I could not take off (…). An excellent nurse took a paper, a pen, and she wrote all the alphabet letters. She showed me the letters to compose the word… (P9)

(…) I could not speak, and they [HCPs] gave me a sheet and a pen with which to write what I wanted, what I could do. (P16)

###### 3.2.2.1.2 Sub‐Theme 5.2 Communication Between Professionals and Patients' Families

Some participants recounted that their family members received regular telephone updates from physicians about their health condition, including critical decisions such as intubation.

From what I have heard and what my wife has told me, the physicians on duty regularly called her twice a day. (P1)

I had heard that she [the daughter] always called the ward to get news. Even the day they were going to intubate me, they called her and told her it was probably going to happen… they did a good job of getting the whole family involved (…). (P5)

(…) every day, without fail, the physicians called my children and my husband. (P10)

###### 3.2.2.1.3 Sub‐Theme 5.3. Using Technology to Communicate With Loved Ones

Some patients highlight the use of technology as a positive strategy useful in limiting the isolation of patients from their loved ones. Some participants emphasised the proactive role of nurses who provided their own devices to show videos of the patient's family members or to make video calls facilitated by them.

In intensive care unit, I remember that a nurse with a cell phone, perhaps from the ward, showed me videos that were sent [by family members] for me. (P20)

When I was in intensive care unit, we had a tablet available (…). They [HCPs] agreed with my family, they prepared it [the tablet] and started the video call (…). (P26)

## 4 Discussion

The study analysed memories of the experiences of SARS‐CoV2‐infected patients admitted to ICU about their experiences of care and interaction with HCPs, and their experiences of communication and connection with the outside world.

Regarding demographics, our study sample is gender representative of the general population admitted to intensive care for severe SARS‐CoV‐2 infection. In fact, it is composed to a greater extent of males, who have a higher risk of being transferred to the ICU for the management of severe complications and represent a higher percentage of deaths (Peckham et al. 2020), also in the Italian territory (Quaresima et al. 2021).

Regarding patients' experiences of care and interaction with HCPs (Category a), four themes emerged. In general, the narratives of the interviewed patients highlight a largely positive experience of the received care, although Italy was the first European country to be affected by the pandemic, and its national health service was suddenly faced with a major crisis (Bosa et al. 2022).

Concerning the theme 1, patients told about their experiences of a high level of professionalism and humanity from the HCPs during their stay in the ICU. They recognize the skill and dedication of all HCPs to provide critical care in a difficult situation by reporting that they received excellent and quality assistance. Participants expressed appreciation for the health professionals who helped them, not only for their technical skills but also for their interpersonal skills. This finding is in line with another similar study that shows patients' appreciation for the compassion and competence of HCPs (Kürtüncü et al. 2023). In addition, patients show awareness of the rapidly changing treatment scenario for COVID‐19 influenza. Patients recognize the presence of differences in treatment protocols over time and the lack of established protocols at the beginning of the pandemic. This is in line with what happens when dealing with a new virus, where treatment modalities are constantly being refined based on emerging studies (Jirjees et al. 2021). Patients also tell of uncertainty about the best course of action to follow, highlighting the need for clear and consistent communication about treatment options and evolving knowledge.

Such perceptions are consistent with the literature, particularly in the early stages of the pandemic when COVID‐19 was an unknown disease and there were frequent changes in treatment and care approaches by HCPs (e.g., medication, use of mechanical ventilation and supination in the ICU) (Chua et al. 2021; Azoulay et al. 2020).

Theme 2 presents the challenges faced by ICU patients during the pandemic. Their experiences mostly relate to the problems faced during critical care. The use of PPE emerged as a barrier to the quality of care. Patients recounted difficulties in communication due to the fogging of visors and the discomfort felt by professionals that even caused one physician to faint. These findings are in line with previous literature by documenting the difficulty due to PPE, including impaired communication, reduced agility and stress caused by heat (Kürtüncü et al. 2023; Ozan and Durgu 2023; Duan et al. 2021), which may result in serious issues during extended shifts. Patients' narratives tell about a HCPs shortage during the pandemic, which is also reported in the literature (Clari et al. 2021), together with the critical need to train a proportion of nurses to deal with the high number of patients in isolation or intensive care (Bergman et al. 2021). Although the patients highlight a situation in which the intensive care unit was adequately equipped to deal with the critical situation, they tell about overworked and exhausted HCPs. Professionals were ‘suffocated’ due to lack of staff. This is in line with what emerged from the reports on burnout and shortage of healthcare professionals during the peak of the pandemic (Damico et al. 2020; Mushtaq et al. 2022). Understaffing inevitably leads to the neglect of nursing interventions. In fact, the COVID‐19 pandemic has affected nursing interventions (e.g., education) and the nurse–patient relationship (Ozan and Durgu 2023; Smith and Farra 2022), due to the necessary infection prevention measures (Clari et al. 2021; Zipf et al. 2022). This leads to limiting interventions to those needed to address urgent clinical and nursing issues. As a result, care moves away from its holistic view, and this can increase barriers that lead to dehumanisation of care (Fernandes et al. 2022).

Another crucial aspect that emerged from the patients' narratives concerns the absence of a post‐COVID care pathway, which may have hindered patients' recovery. Patients felt abandoned after discharge, highlighting a lack of continuity of care or support to reduce the long‐term effects of the virus. This gap in the health care system's response to the pandemic, with long‐term care programs for patients, was also emerged in previous research that highlights the prevalence of long‐Covid symptoms (Piras et al. 2022; Carfì et al. 2020).

Although patients generally understood that the reason behind the use of PPE was about the safety of professionals, theme 3 suggests that PPE created a relationship barrier and difficulty in identification, thus creating a sense of anonymity towards professionals. This kind of barrier could potentially hinder the development of a caring relationship as a factor favoring positive patient outcomes (Torun et al. 2023; Hampton et al. 2020).

Communication is a fundamental component of the nurse–patient relationship and an essential aspect of professional practice. Establishing an appropriate nurse–patient relationship should also be a priority in ICUs to ensure humanised care. The participants reported feeling lost and confused when interacting with professionals wearing PPE and being unable to distinguish one from another. However, patients told about professionals implementing interventions (e.g., displaying name and profession on the suit) to provide humanised care to them and establish an effective interpersonal relationship. Introducing new methods (e.g., introducing oneself by defining one's role, carrying a photo showing one's face without a mask, communicating non‐verbal information verbally using body language) makes it easier for patients to recognise professionals (Fernandes et al. 2022; Marler and Ditton 2021) and overcome physical barriers by improving the interaction with patients.

The narratives show that in this particular care situation, patients were seeking a different kind of connection with professionals. Facial expressions, a vital component of nonverbal communication, were hidden due to the PPE. This could potentially lead to misunderstandings, reduced empathy and difficulty in accurately assessing patients' needs. However, some patients found alternative ways to make contact, focusing on eye contact. This underscores the importance of providing training on nonverbal communication for professionals working with PPE to ensure clear and effective communication with patients. Despite difficulties, patients reported being able to perceive professionals' emotional signals through gaze and gestures. This finding underscores the power of eye contact in creating emotional connections even when facial expressions are hidden. In fact, the interviewees reported that the professionals' eyes ‘conveyed serenity’ and ‘smiled’. Research suggests that eye contact increases feelings of trust, relationship and empathy (Gualandi et al. 2023). These emotional connections can be crucial to patient well‐being.

Results from Theme 4 show that patients developed strong emotional bonds with nurses, seeing them almost as family members. In fact, nurses are perceived not only as professionals, but also as people who convey human closeness through gestures and behaviours. Nurses are referred to as ‘relatives’ or ‘brothers’ or other terms used to describe close people who would normally care for them. This is in line with the study by Torun et al. (Torun et al. 2023), in which patients made positive analogies with nurses considering them ‘angels’ and their ‘relatives’. This is in line with previous research on ICU patients, where social isolation and limited family contact are prevalent (Køster et al. 2023). Nurses, being the main source of human interaction and care, become crucial emotional support systems, thus filling the void left by the physical absence of family. Patients' narratives emphasise the development of a unique and ‘special’ bond with nurses. This is consistent with previous research suggesting that ICU patients appreciate nurses' constant presence during a difficult time and report feeling better when they are cared for (Cederwall et al. 2018; Wassenaar et al. 2014).

Regarding patients' experiences of communication and connection with the outside world (Category b), Theme 5 examines the topic of communication and the strategies offered to make it possible inside the ICU and with patients' families. Specifically, patients explained that communication between patient and practitioner, because of the limitations in verbal communication (e.g., breathing difficulties, use of breathing equipment), occurred through nonverbal signals such as gestures and nods, thus highlighting the crucial role of nonverbal communication (Chua 2022). Some patients report acceptance of creative solutions implemented by professionals, including the use of written communication aids such as alphabet boards and notepads. This is consistent with findings in the literature on the use of alternative communication methods in the ICU (Akgün et al. 2020). Participants reported that their family members regularly received telephone updates from physicians about their clinical conditions. Information about the ongoing involvement of relatives in the care process emerges as a positive element in the stories. This finding is in line with previous research suggesting that frequent and clear communication with families is essential to reduce anxiety and increase confidence (Cederwall et al. 2018; Akgün et al. 2020). In addition, to reduce the distance between patients and their families, a strategy was the use of technology. Patients recount that nurses provided personal devices to show patients videos from their loved ones or facilitated video calls. These practices can improve patient‐family communication and increase the emotional well‐being of ICU patients, especially during pandemic restrictions on visits (Kennedy et al. 2021; Jungestrand et al. 2023).

### 4.1 Limitations

This study had limitations that should be considered in future research. The qualitative nature of the data makes it difficult to generalise the results, which should be used with caution. In fact, these results are based on a small sample size and are not representative of the population of patients admitted to the ICU at the time of COVID‐19.

The female gender is less represented in relation to the sample. However, it should also be considered that in Italy the male gender has a higher risk of being admitted to the ICU due to the more severe complications to be managed. With regard to the sample, a limitation is that the patients interviewed may not be fully representative of the ‘ordinary’ ICU patient during the pandemic, because they were recruited after spontaneous interviews and public statements about their experience. In this sense, they may be better able to respond to critical events than other people who have experienced a similar situation.

Another limitation concerns the fact that in the ICU, patients may have an altered state of consciousness, especially when they are in severe pain and are being treated with drugs that keep them sedated. This could affect the authenticity of the data. Moreover, the study examines the participants' memories of their experience during their admission to the ICU, but it may be the overall experience of the hospital stay that is most remembered. It should be kept in mind that the results may have been influenced by the emotional or physical condition of the respondents at the time of the interview, or even by the dominant memory of the hospital stay. In fact, the study was conducted during the first waves of the COVID‐19 pandemic, when Italy was the first country in Europe to reorganise its health system to respond to the health emergency. In addition, most of the respondents were hospitalised in the areas most affected by the pandemic in Italy.

### 4.2 Implications and Future Research

The study results offer interesting insights for improving practice in intensive care units and patient care in the event of future pandemics. The importance of improving communication about treatment options and continuity of care pathways after hospitalisation is emphasized. The development of standardized post‐acute care programs focused on patient needs can ensure continuity of care and improve long‐term adverse outcomes. Improved comfort and functionality of PPE is suggested to overcome communication limitations and address psychological needs. Strategies to address patients' potential sense of isolation and confusion due to PPE barriers could be useful. These could include recognizing the value of technology by providing patients with whiteboards or other communication tools to express themselves and encourage a more patient‐centered approach to care. Future research could address the impact of PPE on practitioners' communication experiences to understand the challenges they faced and their perspectives on the quality of methods used in critical care.

In light of these findings, further studies could investigate how nurses can implement targeted interventions to improve perceived quality. Even in extreme care situations, such as during a pandemic, nurses should always provide the most personalized care possible. Therefore, the treatment of a patient's condition should never result in an ineffective human approach to care.

## 5 Conclusions

This study found that despite barriers due to safety devices, patients with COVID‐19 felt supported and cared for by healthcare professionals in several aspects during hospitalisation, but post‐discharge care programs are needed to reduce the long‐term effects of the disease and provide more patient‐centered care in the ICU and during future outbreaks.

## Author Contributions

Ilenia Piras: Conceptualization, Formal analysis, Writing original draft, Supervision. Maria Francesca Piazza: Formal analysis, Writing original draft, Critical review and editing. Marta Sarritzu: Data collection, formal analysis, writing original draft. Cristina Piccolo: Data collection, formal analysis, writing original draft. Gabriele Finco: Writing original draft, Supervision, Critical review and editing. Maura Galletta: Conceptualization, Supervision, Writing original draft, Critical review and editing.

## Conflicts of Interest

The authors declare no conflicts of interest.

## Data Availability

The data that support the findings of this study are available on request from the corresponding author. The data are not publicly available due to privacy or ethical restrictions.
